# The problematic syndrome of right temporal lobe atrophy: Unweaving the phenotypic rainbow

**DOI:** 10.3389/fneur.2022.1082828

**Published:** 2023-01-09

**Authors:** Christopher R. S. Belder, Anthipa Chokesuwattanaskul, Charles R. Marshall, Chris J. D. Hardy, Jonathan D. Rohrer, Jason D. Warren

**Affiliations:** ^1^Department of Neurology, National Hospital for Neurology and Neurosurgery, Queen Square, London, United Kingdom; ^2^Adelaide Medical School, The University of Adelaide, Adelaide, SA, Australia; ^3^Division of Neurology, Department of Internal Medicine, King Chulalongkorn Memorial Hospital, Thai Red Cross Society, Bangkok, Thailand; ^4^Dementia Research Centre, Department of Neurodegenerative Disease, UCL Queen Square Institute of Neurology, University College London, London, United Kingdom; ^5^Preventive Neurology Unit, Wolfson Institute of Population Health, Queen Mary University of London, London, United Kingdom

**Keywords:** right temporal lobe atrophy, semantic dementia, frontotemporal lobar degeneration, primary progressive aphasia, social cognition, behavioral variant frontotemporal dementia (bvFTD)

## 1. Introduction: The problem

The frontotemporal dementias (FTD) are a clinically and neurobiologically diverse group of diseases that collectively constitute a major cause of younger onset dementia (1–3). Three canonical clinico-anatomical syndromes of FTD are currently recognized: non-fluent variant primary progressive aphasia (nfvPPA), characterized by impaired language output, with predominant anterior left peri-Sylvian atrophy; semantic variant primary progressive aphasia (svPPA), characterized by impaired understanding of words, objects, concepts and socio-emotional signals, with predominantly left-sided antero-mesial temporal lobe atrophy; and behavioral variant frontotemporal dementia (bvFTD), characterized by impaired behavioral regulation and disordered inter-personal conduct and awareness, with a variable atrophy profile. The syndromes of bvFTD and svPPA have an important interface of clinical and neuroanatomical overlap, with a key locus in the right anterior temporal lobe: patients with selective or disproportionate right temporal lobe atrophy (RTLA) may have a distinctive clinical syndrome spanning the bvFTD-svPPA interface, however encapsulating this syndrome has proved both challenging and controversial (4–6).

Here, we argue that defining the RTLA syndrome will depend ultimately on the answers to three key questions. Is a new syndromic category of RTLA justified? If so, what are its core features? And is the syndrome of RTLA truly native to the right temporal lobe? We offer a response to each of these questions, and suggest next steps to consolidate the syndrome and resolve the issues it raises.

## 2. Is a new syndromic category of RTLA justified?

Clinical experience suggests that there is indeed a distinctive syndrome of RTLA, but despite a now substantial (and growing) literature [for example (5–8)], a coherent definition that demarcates this syndrome from related neurodegenerative syndromes remains elusive. Patients presenting with RTLA frequently show strikingly impaired understanding of other people's emotional states and very prominent behavioral features, including lack of empathy, inflexibility, obsessionality, food faddism, loss of libido, disinhibition and apathy (5–8). Impaired recognition of familiar faces (prosopagnosia) often develops, despite well preserved perceptual and other cognitive skills. All of these clinical features also manifest in bvFTD and/or svPPA: it is their conjunction and early salience which tend to distinguish patients with RTLA. The clinically significant way-finding difficulty that sometimes accompanies RTLA superficially resembles the topographical disorientation of Alzheimer's disease but may reflect impaired recognition of topographical landmarks, potentially also encompassing ‘landmark' events in patients' personal timelines (9). Based on a detailed clinical, neuropsychological, neuroanatomical, neuropathological and genetic analysis of 46 cases, Younes et al. (4) have recently proposed that this syndrome be designated “semantic behavioral variant frontotemporal dementia”, with core diagnostic criteria of loss of empathy, difficulty identifying familiar people and complex compulsions or mental rigidity (4).

The demarcation of the RTLA syndrome from svPPA is particularly pertinent. These syndromes lie on a clinical and neuroanatomical continuum – their signatures increasingly converge with disease evolution (10–13), and right temporal lobe involvement occurs early in even typical svPPA (14). In both syndromes, pathogenic protein spread targets a core, bi-temporally distributed semantic appraisal network (10, 15–17). Furthermore, there is a strong, joint association with TAR-DNA-binding-protein (TDP)-43 type C as the underlying proteinopathy (3, 6, 11), albeit with greater genetic and histopathological heterogeneity in RTLA (18, 19).

## 3. What are the core features of the RTLA syndrome?

Complex and profound affective and behavioral disturbances are integral to the RTLA syndrome (4, 5), and these features have yet to be fully characterized. Empathy may, for example, be misplaced and/or caricatured rather than blunted (20). Certain features such as religiosity, musicophilia, obsessions around puzzles, colors or time-keeping, somatising and other odd (sometimes synaesthetic) sensory experiences, while still quite sketchily described, appear to be quite specific for RTLA set against other cases of bvFTD or svPPA (4–7, 21–23). Moreover, along with more pervasive disturbances of affect, humor, social awareness, pain sensibility, appetite and circadian rhythms, these behavioral changes often occur early, even well before the onset of cognitive deficits such as prosopagnosia (19, 23–28). This is a bewilderingly heterogeneous clinical constellation, and unlikely *a priori* to be underpinned entirely by a primary semantic deficit. However, we presently lack standardized tests and metrics to characterize such complex behavioral functions and they do not form part of most neurological and neuropsychological assessments.

While the right anterior temporal lobe mediates socio-emotional concepts (29, 30), the cognitive and neural architecture of such concepts has not been delineated. Emotion recognition deficits are not accounted for by standard measures of semantic competence (31). Even recognition of faces (probably the best characterized nonverbal semantic category) is likely on computational grounds to depend on pre-semantic tagging of configurational salience and familiarity, and thus to engage affective mechanisms (32). Moreover, while theory of mind and empathy – the paradigmatic operations of human social cognition – appear to depend critically on the integrity of right anterior temporal structures, mentalising deficits are not prefigured by semantic impairment (33, 34).

A physiological perspective may be needed to identify common threads that bind the diverse clinical symptomatology of RTLA together. One candidate pathophysiological driver is abnormal reward coding (35): many affective and behavioral changes accompanying RTLA could be interpreted as a shift of hedonic valuation away from other people and toward alternative (sometimes abstract or bizarre) inanimate targets. This hedonic reorientation might in turn reflect impaired integration of interoceptive homeostatic and external sensory signals, also accounting for the autonomic and somatic symptoms experienced by many patients with RTLA. This formulation assigns to the right anterior temporal lobe a core ‘appraisal' role within the semantic appraisal network, in line with previous evidence in the healthy brain and in various diseases states (33, 36, 37). Further, it is informed by currently available (albeit limited) neurophysiological evidence indicating that RTLA is associated with impaired interoceptive awareness and reduced facial emotional micro-reactivity (38, 39). The latter constitutes a physiological signature of the striking ‘poker face' that often signals RTLA in the clinic. However, current clinical tests and metrics do not adequately capture alterations in complex hedonic and homeostatic functions.

## 4. How “right temporal” is the syndrome of RTLA?

**Figure 1:**
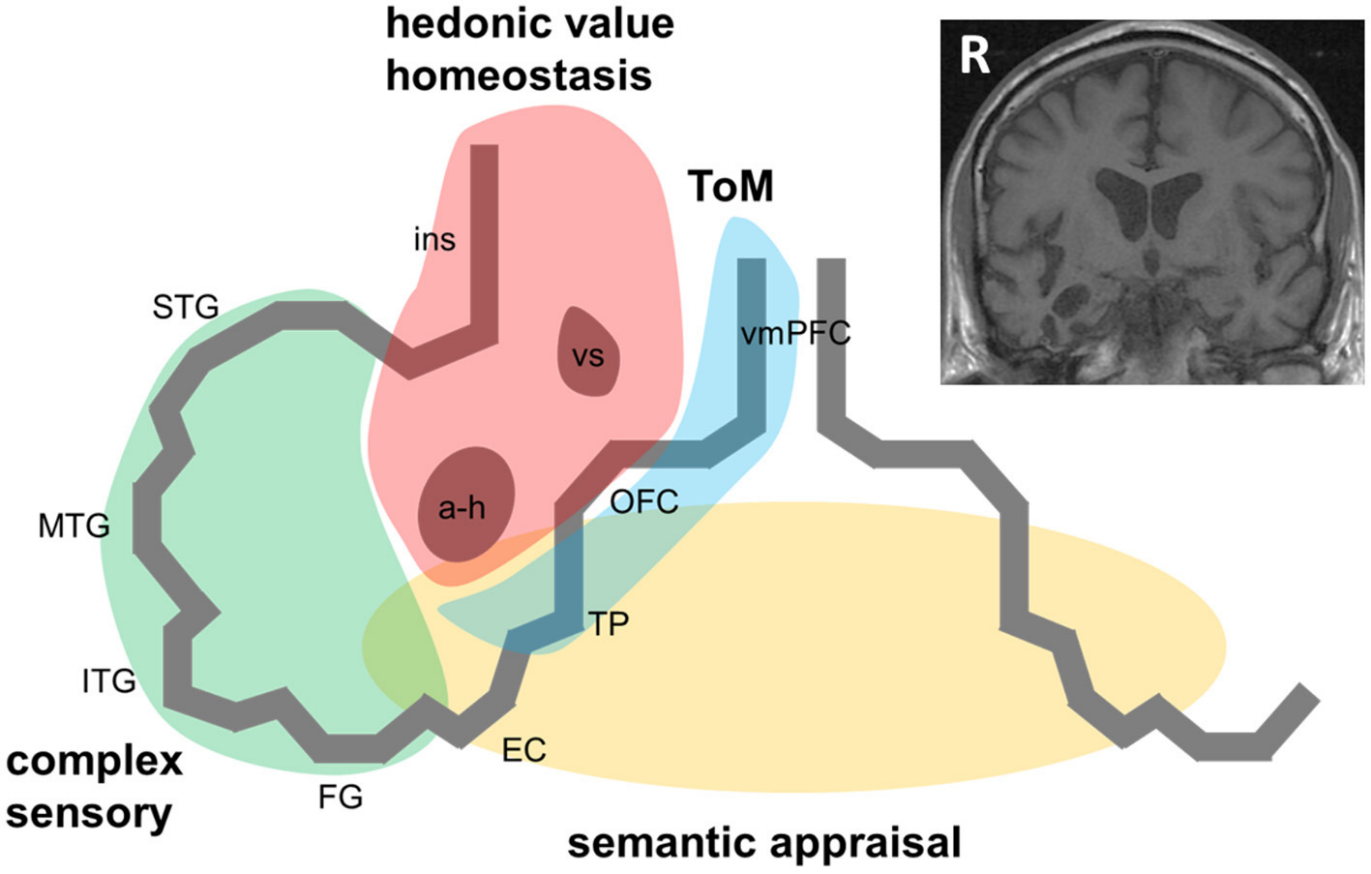
A pathophysiological schematic of the syndrome of right temporal lobe atrophy. The Figure diagrams key brain regions, networks and cognitive processes implicated in the pathogenesis of the syndrome of right temporal lobe atrophy (RTLA). The main cartoon is oriented following radiological convention with the right temporal lobe projected on the left, as in the inset coronal T1-weighted structural brain MRI section (derived from a patient with a clinical syndrome of RTLA). We argue that the clinical syndrome of RTLA reflects the intersection of four large-scale neural networks in the anterior, inferior and mesial right temporal lobe, each mediating a cognitive process that is core to the expression of the RTLA syndrome: complex sensory analysis (green); homeostasis and assignment of hedonic value (red); semantic appraisal of sensory signals (gold); and inference about and response to others' mental states, mentalising or ‘theory of mind' (blue). Note that each of these networks extends beyond the right temporal lobe, to the left temporal lobe and beyond. The cognitive processes mediated by these networks interact extensively; these interactions are likely to fundamentally underpin the diverse phenotypic repertoire of RTLA. Although the core functional neural circuit lesion of RTLA has not been defined, a plausible candidate (relevant to all the cognitive operations schematised here) is impaired neural template-matching (see text). a-h, amygdala and hippocampus; EC, entorhinal cortex; FG, fusiform gyrus; ins, insula; ITG, inferior temporal gyrus; MTG, middle temporal gyrus; OFC, orbitofrontal cortex; R, right; STG, superior temporal gyrus; ToM, theory of mind; TP, temporal pole; vmPFC, ventromedial prefrontal cortex; vs, ventral striatum.

Case ascertainment in most studies of the RTLA syndrome has been based on MRI evidence of right temporal lobe involvement disproportionate to atrophy of the adjacent frontal lobe or contralateral temporal lobe. Applying this criterion does tend to differentiate cases with RTLA syndromically from other cases of bvFTD with right temporal lobe involvement (8). A profile of antero-mesial and inferior temporal cortical atrophy (with accompanying regional hypometabolism) that “mirrors” the more common profile of svPPA in the left temporal lobe seems most likely to present with the RTLA syndrome [(5–8); Figure 1]. However, it is not clear that all patients even with this selective atrophy profile have the same clinical syndrome; indeed, in clinical practice, semiotic diversity is itself a signature of RTLA cases.

Like all neurodegenerative proteinopathies, RTLA is a neural network-based disease: it remains uncertain to what extent the syndrome of RTLA depends on conjoint involvement of (or disconnection from) other network elements, both in the contralateral temporal lobe and in ipsilateral, more posterior temporal, insular and inferior frontal cortices and subcortical structures [(4, 10, 13, 14, 16, 17, 33, 37); Figure 1]. This will only be resolved by detailed longitudinal correlation of clinical features with evolving network dysfunction and atrophy, quantified using volumetric MRI, voxel based morphometry, tractography and functional MRI. A related puzzle is the comparative rarity with which proteinopathies such as TDP-43 initially strike the right compared with the left temporal lobe (3, 11): while this could in part reflect ascertainment bias (right-sided presentations are more likely to be overlooked or misattributed to a psychiatric process than language presentations), it may be telling us something of fundamental importance about the relative susceptibility of neural circuitry in the left and right temporal lobes to neurodegeneration.

Symptoms of RTLA – particularly those reflecting abnormal evaluation of sensations arising from one's own body, extended representation of the self in time and decoding the emotional signals of other people (33, 37, 38, 40, 41) – may arise from dysfunction of distributed, connected brain regions beyond the right temporal lobe. According to this formulation (diagrammed in Figure 1), the right anterior temporal lobe acts as a “hub” at the interface of semantic appraisal, mentalising, reward and autonomic control networks. This circuitry is essential to the social brain connectome (33).

How many syndromes of RTLA are there? It has been proposed that there are at least two, neuroanatomically separable syndromes associated with RTLA (8), with scope on genetic and histopathological grounds for additional sub-syndromes (18, 19).

## 5. Discussion and future view

The symptomatology of RTLA presents a veritable rainbow of cognitive and behavioral deficits; as with any rainbow, unpicking the spectrum entails a risk of missing its essence. Pragmatically, does it really matter what a syndrome is called? We contend that it does, because nosology matters: it guides diagnosis (and reduces misdiagnosis), shapes research agendas, and determines how diseases are interpreted for patients and families, and how they are managed. The syndrome of RTLA exemplifies a broader, topical controversy in the field of neurodegenerative disease: namely, whether and how dementia syndromes should be segmented, in the face of high phenotypic variation (42). As the histories of bvFTD and PPA attest, defining syndromes using clinical diagnostic criteria can have considerable value in motivating research to elucidate underlying pathophysiology. The RTLA syndrome, however, poses the conundrum of clinical phenomena that are perhaps uniquely challenging to operationalise.

We argue that any re-conceptualization of the RTLA syndrome must await a fuller characterization of the mechanisms whereby RTLA wreaks its hedonic and homeostatic effects, with histopathological and molecular correlation (6, 19). As the culprit pathologies are individually rare, this will entail collaboration between specialist centers, each implementing a uniform assessment protocol and collecting correlative neuroanatomical and neuropathological data. However, cooperative enterprises of this kind will depend on a shared framework for defining the syndrome - as is amply endorsed by the work of Younes et al. (4), Ulugut Erkoyun (5), and Campos et al. (6). Any such framework should ideally be informed by a deeper understanding of how social and emotional concepts and reward value are represented in the healthy brain – and how these are modulated according to sensory experience, homeostatic state and behavioral goals. Indeed, RTLA appears to be an ideal “lesion model” for identifying critical attributes of the cognitive and neural architecture that links socio-emotional concepts, nonverbal semantics and reward more generally (17, 30, 43). Relatedly, there is a need to deconstruct complex, multi-dimensional behavioral symptoms such as “obsessionality”, “disinhibition”, and “apathy”.

Here we have suggested that disintegration of the normal linkage between homeostatic, affective and semantic circuits could produce a fundamental shift in hedonic valuation away from inter-personal and toward inanimate or abstract targets, manifesting as the diverse and often bizarre preoccupations that tend to signal RTLA. Although the core functional neural circuit lesion of RTLA has not been defined, one plausible candidate is impaired neural template-matching, manifesting as inappropriate behavioral responses to inter-personal and environmental signals. Neurophysiologically, this could arise from degraded short- and longer-range interneuronal inhibitory connections (16, 20, 36). Testing this experimentally would require dynamic techniques that can capture alterations in neural network connectivity. This could be achieved by integrating multimodal approaches that measure interoceptive and exteroceptive reactivity, affective and semantic decision-making and neural network functional anatomy (16, 43). As part of this enterprise, it will also be important to address such striking but poorly understood phenomena as religiosity, musicophilia and color obsessions, which may hold a key to defining the syndrome of RTLA.

Clinically, we currently lack the tools to fully define the syndrome of RTLA – conventional neuropsychological tests give an incomplete picture. We may need to supplement current batteries with new tests of social and emotional cognition and physiological markers of hedonic and homeostatic function. We argue that only a pathophysiologically informed, collaborative, prospective and longitudinal analysis of the RTLA syndrome will allow its core to be defined – thereby guiding the development of bespoke clinical tests and markers, which are likely to transcend currently standard approaches.

## Author contributions

CB and JW initiated the project and prepared the first draft of the manuscript. AC, CM, CH, and JR made intellectual contributions. All authors critically reviewed and approved the submitted paper.
